# Wearable sensors for monitoring the physiological and biochemical profile of the athlete

**DOI:** 10.1038/s41746-019-0150-9

**Published:** 2019-07-22

**Authors:** Dhruv R. Seshadri, Ryan T. Li, James E. Voos, James R. Rowbottom, Celeste M. Alfes, Christian A. Zorman, Colin K. Drummond

**Affiliations:** 1 Department of Biomedical Engineering, Case Western Reserve University, 10900 Euclid Avenue, Cleveland, OH 44106 USA; 2 Department of Orthopaedic Surgery, University Hospitals Cleveland Medical Center, Cleveland, OH 44106 USA; 3 University Hospitals Sports Medicine Institute, Cleveland, OH 44106 USA; 4 Department of Cardiothoracic Anesthesiology, The Cleveland Clinic, 9500 Euclid Avenue, Cleveland, OH 44195 USA; 5 Frances Payne Bolton School of Nursing, Case Western Reserve University, 9501 Euclid Avenue, Cleveland, OH 44106 USA; 6 Department of Electrical Engineering and Computer Science, Case Western Reserve University, 10900 Euclid Avenue, Cleveland, OH 44106 USA

**Keywords:** Diagnostic markers, Predictive medicine

## Abstract

Athletes are continually seeking new technologies and therapies to gain a competitive edge to maximize their health and performance. Athletes have gravitated toward the use of wearable sensors to monitor their training and recovery. Wearable technologies currently utilized by sports teams monitor both the internal and external workload of athletes. However, there remains an unmet medical need by the sports community to gain further insight into the internal workload of the athlete to tailor recovery protocols to each athlete. The ability to monitor biomarkers from saliva or sweat in a noninvasive and continuous manner remain the next technological gap for sports medical personnel to tailor hydration and recovery protocols per the athlete. The emergence of flexible and stretchable electronics coupled with the ability to quantify biochemical analytes and physiological parameters have enabled the detection of key markers indicative of performance and stress, as reviewed in this paper.

## Introduction

Biomedical sensors present an exciting opportunity to measure human physiologic parameters in a continuous, real-time, and nonintrusive manner by leveraging semiconductor and flexible electronics packaging technology.^1^ These sensors incorporate a broad range of advances in microelectromechanical (MEMS),^2^ biological and chemical sensing,^3^ electrocardiogram (ECG),^4^ electromyogram (EMG),^5^ and electroencephalogram (EEG)-based neural sensing platforms.^6^ Biological and chemical sensors are increasingly viewed as promising alternatives to expensive analytical instruments in the health care industry when specificity and selectivity criteria are met. The development of electrochemical transducers has been especially promising due to their low cost, simplicity, and portability.^7,8^ This has led to the development of commercial hand-held sensors, such as ACCU-CHEK® by Roche Diagnostics, iSTAT® by Abbot, and Lactate Scout® by Sports Resource Group for the measurement of metabolites and electrolytes.^3^ However, these sensors require blood samples thus posing as a barrier to their utility in real-time monitoring for sports medicine. The emergence of wearable biosensors to measure analytes from eccrine sweat to assess the performance and mental acuity of the athlete serve as next steps to assessing human performance. This review discusses the application of wearable sensors to measure analytes from saliva and eccrine sweat affecting athlete performance and the use of such devices to assess the mental acuity and stress of the athlete based on heart rate variability (HRV), galvanic skin response, and biomarkers measured from eccrine sweat. The discussions in this paper highlight advancements in scientific literature and provide insight into the commercial landscape of this growing field (Tables 1 and 2).

**Table 1:** Sampling of wearable technology companies with products applicable towards measuring biomarkers from eccrine sweat or saliva

Company	Sampling of products	Product type	Product functionality	Headquarters
BSX Technologies	LVL	Wrist-based device	Hydration, fitness, heart rate, mood, and sleep	Austin, TX
Eccrine Systems	Sweatronics®	Sweat sensor	Analyte detection from eccrine sweat	Cincinnati, OH
Epicore Biosystems	N/A	Epidermal sensor	Wearable microfluidic sensor to measure lactate, glucose, pH, and chloride ions	Cambridge, MA
Graphene Frontiers	Six™ Sensors	Device unit	Graphene field effect transistor capable of detecting biomarkers, proteins, and amino acids	Philadelphia, PA
GraphWear	GraphWear	Epidermal sensor	Glucose and lactic acid measurements from sweat	San Francisco, CA
Halo Wearables	Halo H1	Wrist-based device	Hydration monitoring	Morgan, UT
Kenzen	Echo H2	Patch	Body temperature, biomarkers (pH, potassium, sodium) to detect hydration, heart rate	San Francisco, CA
Nix	N/A	Hydrogel sensor	Sweat-based biometric sensor to monitor hydration	Boston, MA
Sano	Sano	Patch	Non-invasive glucose measuring	San Francisco, CA
Sixty	Sixty	Wrist-based device	Hydration levels, heart rate, activity levels & calories burnt as well as sleep tracking	Innishannon, Ireland
Xsensio	Xsensio	Epidermal stamp	Energy-harvesting “Lab-on-skin” stamps to detect biomarkers at attomolar concentrations	Lausanne, Switzerland

Data for this table was acquired from company websites and social media sites affiliated with each company

**Table 2:** Sampling of wearable technology companies with products applicable toward measuring the mental acuity and stress of the athlete

Company	Sampling of products	Product type	Product functionality	Headquarters
Bellabeat	Leaf Urban, Leaf Impulse, Leaf Chakra	Smart Jewelry	Relates breathing to stress intensity	San Francisco, CA
Halo Neuroscience	Halo Sport	Headset	Utilizes neuropriming to increase the excitability of motor neurons to assist with athletic training	San Francisco, CA
Interaxon	Muse	Headband	Signal processing from EEGs to detect stress	Toronto, Canada
Neumitra	Neumitra	Watch	Stress quantification	Boston, MA
Prana	Prana	Waistband	Measures breathing and posture	San Francisco, CA
Sentio	Feel	Wristband	Electrodermal activity, skin temperature, and blood volume pulse	Palo Alto, CA
Thync	Relax, Vibe	Device unit	Lowers stress biomarkers such as alpha amylase and buffers stress response via heart rate variability and skin conductance. Device placed on back of the neck	Los Gatos, CA
VivaLnk	Vital Scout, Fever Scout	Wireless Patch	Detects stress via body temperature, respiration rate, sleep, heart rate variability, activity	Santa Clara, CA
Vinaya	Zenta	Wrist-based device	Optical, bio-impedance, and skin conductivity measurements are translated via machine learning to detect stress	London, UK
WellBe	WellBe Bracelet	Bracelet	Translates heart rate measurements into stress levels; provides prognosis to lower stress	Madison, WI

Data for this table was acquired from company websites and social media sites affiliated with each company

## Value proposition for wearables in sports

Sports teams are continuously searching for opportunities to improve the performance and safety of their athletes to gain a competitive advantage on the field. Over the last decade, time-motion analysis systems such as video recording and computer digitization have been utilized to measure human locomotion and improve sports performance. While these techniques were once state of the art, they were faced with questionable validity of the acquired data, labor-intensive nature of collecting data, manual hand-notation techniques, and the inability to track key metrics such as biosignals, physiological parameters, and biochemicals, all of which provide real-time data pertinent to the health and performance of the athlete. Recent advancements in wearable sensor technology from a device to systems standpoint have provided new avenues to change this paradigm and are currently being implemented by teams worldwide. While beyond the scope of this review, one issue plaguing the wearables field is the translation of the data to create actionable insight in its respective clinical domain. Questions such as “what does one do with the data” or “what does the data mean” have clouded the translational utility of this technology. To circumvent such hurdles, sports teams have recently hired “sports scientists” whose responsibilities (among others) entail disseminating the data acquired from the sensors into metrics comprehendible by coaches, trainers, players, and key opinion leaders in an organization to complement current rehabilitation therapies for the betterment of the athlete’s health and performance. For example, data pertinent to player movement from wearable devices has been used to inform coaches of their players workout load to indicate which players are at a higher risk to suffer a soft-tissue injury or those that should be sidelined to prevent the occurrence or reoccurrence of an injury during high acuity training periods.^9^ The value wearable devices are having in sports to track and correlate player workouts, exertions, and loads to soft-tissue injuries can be traced to an interview given by a coach in the National Football League (NFL) where he affirmed that the use of such technology coupled with insightful analytics and necessary athlete-specific recovery protocols have shown to alleviate soft-tissue injuries in the team over a 2-year period.^9^

The use of wearable sensors for sports is at its infancy, with the majority of devices currently used to measure movement-based parameters such as distance, velocity, and acceleration. There remains a major need to “quantify the athlete” by measuring biochemical markers such as electrolytes, analytes, and neuropeptides all of which are indicative of physical exertion and fitness, fatigue, and mental acuity.

## Sensor applications for sports medicine

### Biochemical composition of the athlete to optimize on-field performance

Electrochemical wearable sensors have received considerable attention recently because of their potential to monitor a wide array of biomarkers in a continuous and non-invasive manner.^3^ The majority of existing wearable devices utilized in the sporting community currently focus on monitoring physical or physiological parameters (e.g., motion, HR, respiration rate, RR). However, such devices, do not permit team trainers and physicians to quantify the biochemical profile of an athlete in a real-time manner with the goal of alleviating soft-tissue injuries, dehydration, or cramping. Proper hydration is key to success as under-drinking can lead to hypohydration and over-drinking can lead to hyponatremia (low-serum sodium concentration).^10^ Over the last decade, significant progress has been made to develop wearable electrochemical sensors that detect biomarkers non-invasively from biofluids such as saliva and sweat, both of which are easily accessible without impeding the performance of an athlete. In this section, we review such wearable devices and discuss their utility as it relates to sports medicine.

#### Saliva

**Fig. 1:**
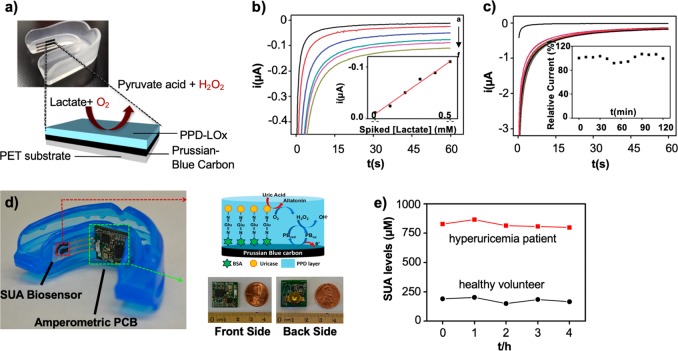
Mouthguard biosensor for the continuous monitoring of metabolites from saliva. **a** Mouthguard biosensor with the integrated printable electrodes. The Prussian Blue working electrode is coated with the PPD-LOX layer for salivary lactate monitoring. **b** Testing of the mouthguard biosensor from (**a**) in human saliva showed that the device responded favorably to changes in lactate level with a correlation coefficient of 0.988. **c** Testing of the mouthguard biosensor from (**a**) to untreated human saliva over a 2-h period demonstrated a highly stable response. The good stability is reflective of the PPD coating against co-existing fouling constituents. **d** Salivary uric acid biosensor with a wireless amperometric circuit board. Chemically modified Prussian-Blue carbon comprised the working electrode. The amperometric printed circuit board (PCB) was the size of a 1 cent coin. **e** Translational utility of the mouthguard demonstrated the ability of the device to measure salivary uric acid levels over a 5-h period in a healthy volunteer (black) vs. that of a patient with hyperuricemia (black). Figures were reproduced with permission from Kim et al.^14^ (**a–c**) and Kim et al. (**d**, **e**).^15^

Saliva is considered an attractive and emerging option compared to direct blood analysis for quantifying biomarkers related to human performance due to its noninvasive nature and continuous supply^11^ (Table 3). In addition, biomarkers detected in saliva such as alpha-amylase, glucose, lactate, phosphate, and uric acid (UA) have been shown to have a good correlation with that found in blood^12^ (Table 4). Initial work pertaining to electrochemical salivary sensors was conducted by Graf in the 1960s to measure pH and fluoride ion levels on a partial denture.^13^ Salivary sensors have been made based on screen-printing manufacturing techniques.^14,15^ Researchers incorporated an amperometric enzymatic biosensor in polymeric mouthguards for monitoring salivary lactate (Fig. 1a–c) and UA concentrations^14^ (Fig. 1d, e). Kim et al. fabricated a mouthguard biosensor to measure lactate levels by screen-printing three separate layers on a flexible polyethylene terephthalate (PET) substrate (Fig. 1a). The first layer comprised of the reference Ag/AgCl electrode, for interfacing to the electrochemical analyzer. The second layer, comprised of the working and auxiliary electrodes, was printed from a Prussian blue-graphite ink. Lactate oxidase (LOx) was coated on the working electrode surface by electropolymeric entrapment in a poly(o-phenylenediamine) film. The third layer was printed by using a dielectric paste and served as the insulator layer. The three printed electrodes were attached to the mouthguard body via a double-sided adhesive. Data from chronoamperograms for increasing concentrations of lactate in phosphate-buffered saline medium showed that the biosensor displayed a very high sensitivity toward lactate, with current-signals proportional to the lactate concentration. The chronoamperometric response of lactate in the presence and absence of physiological concentrations of ascorbic acid and UA showed that these potential interferences had a negligible effect upon the lactate response and that the biosensor system provided high selectivity to measure lactate in a noninvasive manner. Sensor stability was tested over a 2-h period with measurements of 0.05 mM carried out every 10 min. Results from the corresponding chronoamperogram demonstrated a highly stable response over a 2-h duration. Furthermore, the biosensor tested favorably to measuring lactate levels in unstimulated human saliva with good linearity and a correlation coefficient of 0.988 (Fig. 1b). In addition, the biosensor demonstrated stability over a similar 2-h period when treated with unspiked human saliva (Fig. 1c). In another study, Kim et al. fabricated a wearable salivary UA biosensor in a mouthguard (Fig. 1d, e). Mouthguard biosensors were screen-printed on a flexible PET substrate with three layers. The first layer consisted of an Ag/AgCl reference electrode as the current collector. The second layer consisted of a Prussian-blue graphite ink as the reference and counter electrodes. The third layer consisted of a dielectric paste which served as the insulator. Each layer was thermally cured after printing. The working electrode was modified with the uricase enzyme and antibiofouling membranes. The efficacy of the biosensor was successfully assessed in artificial saliva, undiluted human saliva, and in a hyperuricemia patient with and without medication control. The untreated hyperuricemia patient showed a sustained high-SUA level for 5 h (Fig. 1e). Real-time testing of the devices presented are needed to assess the true clinical efficacy of such technology for sports.

**Table 3:** Comparative analysis of biomarker sources towards assessing human performance

Source	Location	Advantages	Drawbacks	SOC	References
Apocrine Sweat	Underarm, groin	Sweat volume, noninvasive and continuous measurements possible	Locations on body may intrude athlete mobility/comfort, limited locations on body.	No. There remains a need to validate fabricated devices in formalized studies	^19^
Blood	In the body	Well validated technology	Cannot be measured continuously, real-time, or noninvasively.	Yes. Sample drawn during physicals or when necessary.	^135^
Eccrine Sweat	Pores distributed across skin (>100 glands/cm^2^)	Noninvasive, continuous measurements possible without intruding on athlete mobility	Skin contamination, dried sweat on the glands, low-sampling rates, sample volume (e.g., function of weather conditions).	Yes. There remains a need to continue to validate wearable devices in formalized studies	^18– 20^
Urine	Bladder	Sample volume, ease of access	Noninvasive, continuous measurements are not possible	Yes, urine color used to assess hydration. Biomarkers from urine used during drug tests	^107, 136, 137^
Saliva	Mouth	Sample volume, ease of access	Limited to sports which require or necessitate mouthguard devices. Point-of-care (POC) devices currently developed in literature do not permit continuous measurements.	No. There remains a need to validate fabricated devices in formalized studies	^12, 14, 15, 107, 108^
Tears	Eyes	Noninvasive measurement possible	Sample volume, continuous measurements not possible over long duration, limited biomarkers can be detected, athlete comfort and safety	No. There remains a need to validate fabricated devices in formalized studies.	^138, 139^

*SOC* standard of care (as defined by their use in sports today)

**Table 4:** Sampling of biomarkers or physiological parameters which have been measured noninvasively from saliva and eccrine sweat sensors for monitoring human performance

Biomarker	Justification to measure biomarker for the athlete	Concentration		Recognition element		Sensing modality	
		Saliva	Eccrine sweat	Saliva	Eccrine sweat	Saliva	Eccrine sweat
Alpha-amylase	Stress levels^140–142^	5–17 U/mL	–	α-Glucosidase, glucose oxidase, mutarotase	–	Biorecognition element	–
Glucose	Fatigue (e.g., hyperglycemia and hyperinsulinemia)^44,59,60,143–145^	1 μM	10–200 μM	Glucose oxidase	Glucose oxidase	Chronoamperometry	Chronoamperometry
Lactate	Workout intensity determined from measuring lactate inflection point^45,47,55,56^	5–50 μM	5–20 mM	Lactate oxidase	Lactate oxidase	Chronoamperometry	Chronoamperometry
Phosphate	Oral health,^146^ uremia^146^	3.6–300 μM	–	Lactate oxidase and Prussian Blue or pyridine-oxazoline	–	Amperometry	–
Na^+^	Hyponatremia^29–31,35,147^	–	10–100 mM	–	Na ionophore	–	Potentiometry
Cl^-^	Fatigue^148^	–	10–100 mM	–	Ag/AgCl	–	Potentiometry
K^+^	Hypo/hyperkalemia^44^		1–18.5 mM	–	K ionophore	–	Potentiometry
pH	Indicative of lactic acid build-up due to increase in [H^+^]^55,149^	–	3–8	–	Polyaniline	–	Potentiometry
NH^4+^	Fatigue; differentiate change from aerobic to anaerobic state^46^	–	0.1–1.1 mM	–	Ammonium ionophore	–	Potentiometry
Orexin A	Cognitive function and stress levels^113^	–	pg–nM	–	ZnO FET	–	Biorecognition element
Cortisol	Cognitive function and stress levels^111^	–	8–140 ng/L	–	ZnO, MoS_2_	–	Electrochemical impedance spectroscopy

Dashed line indicates that the specific biomarker has not been measured using a wearable sensor. Table is adapted and modified with permission from Malon et al.^150^ and Bariya et al.^20^

In the following work, researchers fabricated a MEMS-based sensor designed for the human oral cavity to enable the noninvasive measurement of salivary glucose. The glucose biosensor was comprised of a platinum and silver/silver chloride electrode, with glucose oxidase (GOD) immobilized by entrapment with poly-(MPC-co-EHMA) (PMEH), on a custom-fitted monolithic mouthguard support with a wireless transmitter. The researchers demonstrated the capability of the sensor and wireless communication platform to monitor salivary glucose in a phantom mandible mimicking the environment of the human oral cavity. While a sensor embedded in a cavity may be far removed for sports medicine applications, the work demonstrated the ability to detect glucose, a key marker indicative of fatigue levels. Recently, a collaborative team from PARC, a Xerox Company, NextFlex, and the University of California, San Diego fabricated a smart mouthguard biosensor to detect early signs of dehydration, exhaustion, and mental state based on lactate and glucose measurements from saliva.^16,17^ The sensor was fabricated on a small, flexible plastic foil that was placed on a mouthguard. An encapsulant was applied on top of the sensor to protect it from saliva. Chronoamperometry, based on enzymatic oxidation of the target species, was utilized as the electrochemical detection method. The device enabled replacement of the electrodes to measure other biomarkers such as UA.

While the application of mouthguards to quantify biomarkers is relatively new from a commercial standpoint, there remains a clinical need to validate the sensitivity and reliability of these devices in real-time during athletic scenarios. The main drawback of saliva being used for real-time measurements of human performance compared to eccrine sweat is that it is limited to sports which require mouthguard devices. In addition, analyte concentrations found in saliva are far below those detected in eccrine sweat (Table 4). Furthermore, current devices discussed in literature have not shown continuous measurements of biomarkers from saliva to be possible (Table 3). Developing biosensors with high sensitivity and stability is the initial step to develop these devices for use in sports.

#### Eccrine sweat

Sweat provides an ideal source toward the continuous and noninvasive measurement of biomarkers, such as sodium, chlorine, potassium, lactate, calcium, glucose, ammonia, ethanol, urea, cortisol, and various neuropeptides and cytokines.^18^ Detection of biomarkers from eccrine sweat glands pose tremendous advantages over those from urine, blood, tears, and apocrine sweat glands. These, include their abundance on the body (>100 glands/cm^2^), ease of access, sampling and detection efficiency without foreign contamination during testing, and the inability to degrade analytes^19^ (Table 3). Disadvantages of using eccrine sweat, include skin contamination, existence of dried sweat on the glands thereby skewing analyte measurements, and low-sampling rates.^18^ Wearable devices capable of artificially inducing sweat through the introduction of current (iontophoresis) can overcome hurdles associated with low-collection volumes (e.g., attributed to weather) or skin contamination,^20,21^ as discussed later. However, there remains a need to ensure that the administered current intensity does not cause cathodal vasodilation or erythema, leading to discomfort to the athlete.^22^ Modulating the current intensity and subsequent current density of the epidermis through the use of hydrogel-based electrodes could be a viable first step in alleviating such reactions on the skin. Wearable devices to measure biomarkers from sweat must adhere to the following set of requirements: (1) the sweat analyte must be correlated to those found in blood circulation, (2) the sweat rate must be steady or measured as a result of analyte dilution or sensor-dependencies on sweat rate, (3) sweat must be transported and coupled to sensors in an expeditious manner to minimize analyte exchange with the skin or transport materials themselves, and (4) continuous raw data needs to be shown for the measured analyte in both sweat and blood to ensure that various confounding factors have been resolved (e.g., influence of changing pH or salinity, sensor reversibility, or body motion artifacts).^23^ The need to minimize body motion artifacts are crucial for sports to prevent false sensor readings or cause the sweat flow to accelerate or even reverse. In this section, we highlight the scope of this technology by discussing commercial devices and those presented in recent literature to measure specific analytes crucial to human performance such as sodium, chloride, potassium, lactate, calcium, and glucose (Table 4).

Companies, such as Epicore Biosystems, Halo Wearables, GraphWear, and Kenzen Wear are currently developing epidermal sensors for sweat detection. Epicore Biosystems has established large volume manufacturing for the continuous and noninvasive measurement of various biomarkers from eccrine sweat.^24^ The company has partnered with the Gatorade Sports Science Institute, Seattle Mariners (MLB), US Air Force (USAF), and the Shirley Ryan AbilityLab to further test and validate their devices. Halo H1 has developed the first noninvasive wristband sensor for monitoring hydration levels in athletes by utilizing optical and electrical sensors.^25^ The sensor tracks hydration levels at a cellular level in the bloodstream and utilizes an algorithm to rank the levels out of 100; green (68–100), fully hydrated, yellow (35–67), caution to hydrate soon, and red (1–34), need to hydrate immediately.^25^ GraphWear has fabricated a graphene-based epidermal sensor, which adheres to the torso, to detect glucose and lactic acid for assessing hydration levels.^26^ GraphWear Technologies has piloted their sweat sensor technology with a professional football team.^27^ Similarly, Kenzen Wear’s Echo patch is an epidermal sensor, adhered to the torso, which monitors sodium and potassium from sweat in addition to measuring pH and skin temperature.^28^

#### Sodium and chloride

Sodium (Na^+^) and chloride (Cl^−^) ions are the most abundant electrolytes in sweat. Replacing Na^+^ and Cl^−^ levels after high-intensity situations is instrumental in maintaining electrolyte balance due to their role in stimulating hydration.^29–31^ The total Na^+^ loss from sweat is a function of whole-body (WB) sweating rate and sweat Na^+^ concentration ([Na^+^]); thus, quantifying sodium loss from sweat is vital in expediting player recovery and minimizing soft tissue injuries brought about by the onset of dehydration.^32^ Total whole-body sweat loss and WB sweating rate can be estimated. In the equations presented below EX is during exercise, Pre-ex is pre-exercise, Post-ex is post exercise, WSBL is whole-body sweat loss, and WSBR is WB sweating rate (Eqs (1–3)). Baker et al. developed a model to calculate the WB sweat Na^+^ concentration using absorbent patches from the forearm^33^ (Eq. (3)). Total WB sweat Na^+^ loss can be estimated from total sweat loss and WB sweat [Na^+^] (Eqs (4) and (5)).

1 $${\mathrm{WBSL}}\left({\mathrm{L}}\right) = \left[ {\mathrm{Body}\,\mathrm{Mass}_{\mathrm{Pre} - \mathrm{ex}} - (\mathrm{Body}\,\mathrm{Mass}_{\mathrm{Post} - \mathrm{ex}} - \mathrm{Fluid}\,\mathrm{Intake}_{\mathrm{ex}} + \mathrm{Urine}\,\mathrm{Ouput}_{\mathrm{ex}}}) \right..$$

2 $${\rm {WBSL}}\,\left( {\rm L/h} \right) = {\rm WSBL}/({\rm Exercise}\,{\rm Duration}){.}$$

3 $$\mathrm{Predicted}\,\mathrm{WB}\,\mathrm{Sweat}\left[ \mathrm{{Na}}^ {+} \right]\left( {\frac{{\mathrm{mmol}}}{\mathrm{L}}} \right) = 0.57 \times \left( {\mathrm{forearm}\,\mathrm{sweat}\,\mathrm{Na}^ {+} } \right) + 11.05{.}$$

4 $$\mathrm{WB}\,\mathrm{Sweat}\left[ \mathrm{{Na}}^ + \right]\mathrm{loss}\left( \mathrm{mmol} \right) = \mathrm{WB}\,\mathrm{Sweat}\,\mathrm{Loss} {\ast} \mathrm{WB}\,\mathrm{Sweat}\left[ \mathrm{{Na}}^ + \right]{.}$$

5 $$\mathrm{WB}\,\mathrm{Sweat}\left[ \mathrm{{Na}}^ + \right]\mathrm{loss}\left( \mathrm{mg} \right) = \mathrm{WB}\,\mathrm{Sweat}\,\mathrm{Na}^ + \mathrm{Loss} \ast 22.99\frac{\mathrm{mg}}{\mathrm{mmol}}{.}$$

Sports scientists compared the sweat rates, sweat sodium concentrations, and sodium losses in three groups of NFL players on a single team (backs and receivers [BK], linebackers and quarterbacks [LB/QB], and linemen [LM]) during the second week of two consecutive training camp periods.^34^ Sterile sweat patches were applied to the right forearm after the skin was cleaned and the patches were removed during practice and analyzed via flame photometry. The study showed large variations in sweat Na^+^ concentration between BKs and LB/QB but not between LB/QB and LM. Profuse sweaters required increased dietary consumption of sodium to compensate for such losses during the preseason. This study is significant in that it is the first study pertaining to the quantification of sweat sodium losses to monitor hydration in professional NFL players on a single team utilizing an epidermal patch. A summary of current techniques, challenges, and recommendations used to measure sweat loss and sweat rate are presented in Table 5.

**Table 5:** Summary of current techniques, challenges, and recommendations used to measure sweat loss and sweat rate to assess athlete performance

Current and emerging techniques	Description
Absorbent patches	• Easy to apply, comfortable for the athlete, cost efficient. Worn on locations all over the body (e.g., lower back, forearm, thighs, calf, upper back, forehead) thus permitting measurement from apocrine and eccrine sweat. • Analytically complex, requires baseline sample, time intensive analysis. Accuracy could be cause for concern as eccrine sweat dries on surface
Wearable sensors	• Continuous measurements and actionable insight possible to inform athlete recovery protocols
*Challenges and recommended practices for measuring whole-body sweating rate*	
Varied conditions	• Test conditions (e.g., intensity, environment, and season) specific to athlete’s training and competition • Conduct multiple tests with athletes to determine sweating rate under various conditions
Body mass change (nonsweat)	• Fluid and food intake, respiratory water loss and substrate oxidation, urine output, stool output
Quality control	• Fluid and food intake, respiratory water loss and substrate oxidation, urine output, stool output
*Challenges and recommendations for measuring sweat [Na+] using absorbent patches*	
Varied conditions	• Test conditions (e.g., intensity, environment, and season) specific to athlete’s training and competition • Conduct multiple tests with athletes to determine sweat [Na+] under various conditions
Background contamination methods	• Check for background [Na+] levels and subtract from measured sweat [Na+] values
Skin surface contamination	• Clean skin immediately prior to application and dry with a sodium-free gauze or towel
Anatomical location	• Place in location where maximum sweat can be collected (e.g., lower back)
Adhesion	• Shave area of skin where patch will be adhered • Use appropriate adhesive which will stick to stratum corneum and not cause irritation to the skin
Hidromeiosis	• Limit patch time on the skin and change patches frequently. Use patches with high absorbent capacity. This will help prevent patch saturation
Analysis time	• Transport samples in an appropriate manner to prevent contamination and to inform athletes in a prompt manner to inform and positively effect recovery strategies

The detection of Na^+^ via the use of epidermal sensors has been reported in the literature as well. Bandodkar et al.^35^ reported the successful fabrication and analytical performance of an epidermal tattoo potentiometric sodium sensor for continuous noninvasive monitoring of sodium from eccrine sweat. The screen printed device withstood mechanical deformation without impeding analyte detection and wireless transmission thereby highlighting its translational potential for the sporting community.^35^ Monitoring the change in Cl^−^ concentration in a noninvasive manner along with or independent of sodium measurements utilizing wearables can expedite treatment and recovery to mitigate soft-tissue injuries. While there remains an unmet medical need to measure Cl^−^ levels in real-time for sports, researchers successfully developed a wrist-based potentiometric wearable device capable of detecting Cl^−^ concentrations over time from sweat for cystic fibrosis monitoring.^36^ The sensor was placed on human subjects by a wristband or adhesive tape and tested during exercise to demonstrate the feasibility of this technology as a wearable device. While the use of such adhesion platforms is not appropriate for long-term use in sports, the electrochemical performance and stability of the device demonstrates promise for athletics. In the another study, researchers fabricated a wearable and flexible electrochemical amperometric Na^+^ sensor.^37^ The sensor was composed of a multiwall carbon nanotube (MWCNT) nylon-6 mat resulting in a flexible and conductive sensor. The MWCNTs were functionalized with a cyclo-oligomeric clixarene to selectively form a supramolecular complex with sodium ions. Upon complex formation, the charge carriers migrated from the layer to impede current flow to allow the detection of sodium ions at physiologically appropriate levels for healthy and ailing individuals. In another study, a solid-contact ion-selective electrode and a liquid-junction-free reference electrode were combined together on a dual screen-printed substrate for the detection of sodium from eccrine sweat.^38^ The optimized solid-contact potentiometric strips were integrated with micro-fluidic chips (PotMicroChip) and connected to a passive pump to deliver sweat samples. The system was connected to a miniaturized wireless communications platform entrapped in a 3D printed case to make it wearable. Sodium concentrations were monitored continuously on healthy volunteers during stationary cycling sessions using the device. Comparison of these results to that of current analytical techniques such as atomic absorption spectroscopy, ion chromatography, or commercial sodium meters (e.g., AquaTwin™) would serve as a first step to validate Na^+^ sweat sensors.^38^

#### Potassium

Potassium (K^+^) concentrations in plasma predict muscle activity.^39^ An increase in K^+^ concentration ([K^+^]) during exercise can be explained by the electrical activity in the exercising muscles.^39^ Potassium efflux rate is directly proportional with that of exercise intensity. Potassium is eliminated from the blood by a proportional regulator which may be the Na^+^–K^+^ pump of the exercising muscle.^39^ Extracellular K^+^ is indirectly linked to the pump stimulus and the rate of reuptake is proportional to that of extracellular accumulation. The rate of sweat K^+^ loss has been reported to be indirectly related to sweat flow rate, but the underlying mechanism is unclear and requires further investigation.^40^ Nonetheless, final sweat typically has a [K^+^] similar, albeit with a slightly broader range (~2–8 mmol/L), to that reported for blood plasma.^29,41,42^ Thus, measurement of K^+^ levels could provide tremendous value to gauge and assess the workout intensity, exercise load, and physical exertion of athletes. Xu et al.^43^ devised a low-cost method to mass produce disposable portable sensors for point of care testing of K^+^ from blood serum. The sensor was comprised of poly(3,4-ethylenedioxythiophene) (PEDOT) doped with poly(styrenesulfonate) (PSS) and screen-printed on carbon-based ion-selective and reference electrodes. Polyvinyl chloride (PVC) membranes with and without ionphore valinomycin were coated on the PEDOT/PSS layer to form potassium ion-selective and reference electrodes. The sensor demonstrated a smaller and faster response compared to current standard of care (e.g., clinical laboratory electrolyte analyzer) and required a smaller blood sample volume. However, while such core-sensor technology is promising, it remains impractical for sports medicine. Gao et al. developed a flexible sweat sensor for the real-time detection of Na^+^, lactate, K^+^, glucose, and skin temperature.^44^ Real-time sweat [Na^+^] and [K^+^] measurements were conducted concurrently on six subjects engaged in outdoor running. Sweat [Na^+^] and [K^+^] were deemed stable throughout running in euhydration trials (with water intake of 150 ml per 5 min) after the initial [Na^+^] increase and [K^+^] decrease. An increase in sweat [Na^+^] and a smaller increase in sweat [K^+^] were observed in dehydration trials (without water intake) after 80 min when subjects had lost a large amount of water (~2.5% of body weight). Ex situ measurements of [Na^+^] and [K^+^] from collected sweat samples demonstrated similar phenomena. The researchers hypothesized that this trend was caused by increased blood serum [Na^+^] and [K^+^] with dehydration and increased neural stimulation. We expand on this particular work in the section about glucose measurement from eccrine sweat.

#### Lactate

Lactate and ammonia are small molecules produced during anaerobic activity in the absence of adequate oxygen.^45,46^ Plasma lactate concentrations closely approximate those of sweat lactate and provide an indication of body exertion and exercise intensity.^47^ A hybrid epidermal wearable device comprised of screen-printed three-electrode amperometric lactate biosensors and two-ECG electrodes was fabricated for concurrent real-time measurements of lactate and electrical activity in the heart.^48^ A hydrophobic coating was placed between the two sensor groups to increase the impedance between the ECG and amperometric electrodes thereby preventing crosstalk between the sensor groups. By combining both types of sensors, this wearable device served as a combinatorial platform for physicochemical and electrophysiological monitoring. Real-time monitoring showed that the ECG compared to current wearable devices was not affected by concurrent lactate detection. In addition, lactate levels measured by the biosensor closely approximated the expected sweat-lactate profile for increasing intensity workouts. In another study, a biosensor using luminol as the signaling species was fabricated for lactate detection.^49^ Lactate was oxidized under the catalysis of immobilized lactic dehydrogenase and pyruvate oxidase with nicotinamide adenine dinucleotide as the coenzyme to yield hydrogen peroxide. The formation of hydrogen peroxide enhanced the electrochemiluminescence of luminol thereby permitting the detection of lactate. A detection limit of 8.9 × 10^−12^ mol/L and an average recovery of 101.3% was obtained when utilizing athlete sweat samples during a training course. Adaptation of this device into a wearable could greatly increase its utility for continuous monitoring during training. In another study, a flexible and wearable patch was fabricated to measure lactate, sodium, pH, and temperature.^50^ The sensor was designed to transport sweat via an array of microneedle-type sensors (50 μm diameter) which were incorporated into the microfluidic channel. The potentiometric sodium ion sensors were fabricated using a PVC functional membrane deposited on an electrochemically deposited internal layer of PEDOT. The pH sensing layer was based on a highly sensitive membrane made from iridium oxide. The amperometric-based lactate sensor consisted of doped enzymes deposited on top of a semipermeable copolymer membrane and outer polyurethane layer. A double-layered adhesive was used to secure the 180 μm thick patch to the skin of six healthy subjects prior to cycling and running. Clinical testing showed that perspiration commenced 10–15 min into the warm up period with increasing sweat rate during exercise due to thermoregulation. Sodium and lactate levels increased with an increase in exercise intensity reflecting a rise in anaerobic metabolism. Temperature readings of the sensor varied between 20 and 40 °C across subjects. In the same study, sodium, lactate, and cortisol levels from saliva were detected via various assays. Sodium and lactate levels demonstrated the same correlation as noted with sweat; however, salivary cortisol levels exhibited the largest variation among the subjects. These results suggest that cortisol could be a more sensitive marker for stress, as is discussed later.

#### Glucose

Monitoring glucose levels is crucial for controlling fatigue levels in athletes.^51^ The concentration of glucose in human sweat is in the range of 10–200 µM^20^, and researchers have sought to assess the correlation between blood glucose and sweat glucose levels^52,53^ (Table 4). La Count et al.^54^ modeled the transport of sweat glucose and key electrolyte concentrations to those found in blood. The glucose model, calibrated under a variety of experimental conditions including electrical enhancement, demonstrated a ten-minute blood-to-sweat lag time and a sweat/blood glucose level ranging from 0.001 to 0.02, depending on the sweat rate. Understanding lag times and transport kinetics is key to developing biosensors to accurately measure analytes such as glucose which affect the performance of athletes.

**Fig. 2:**
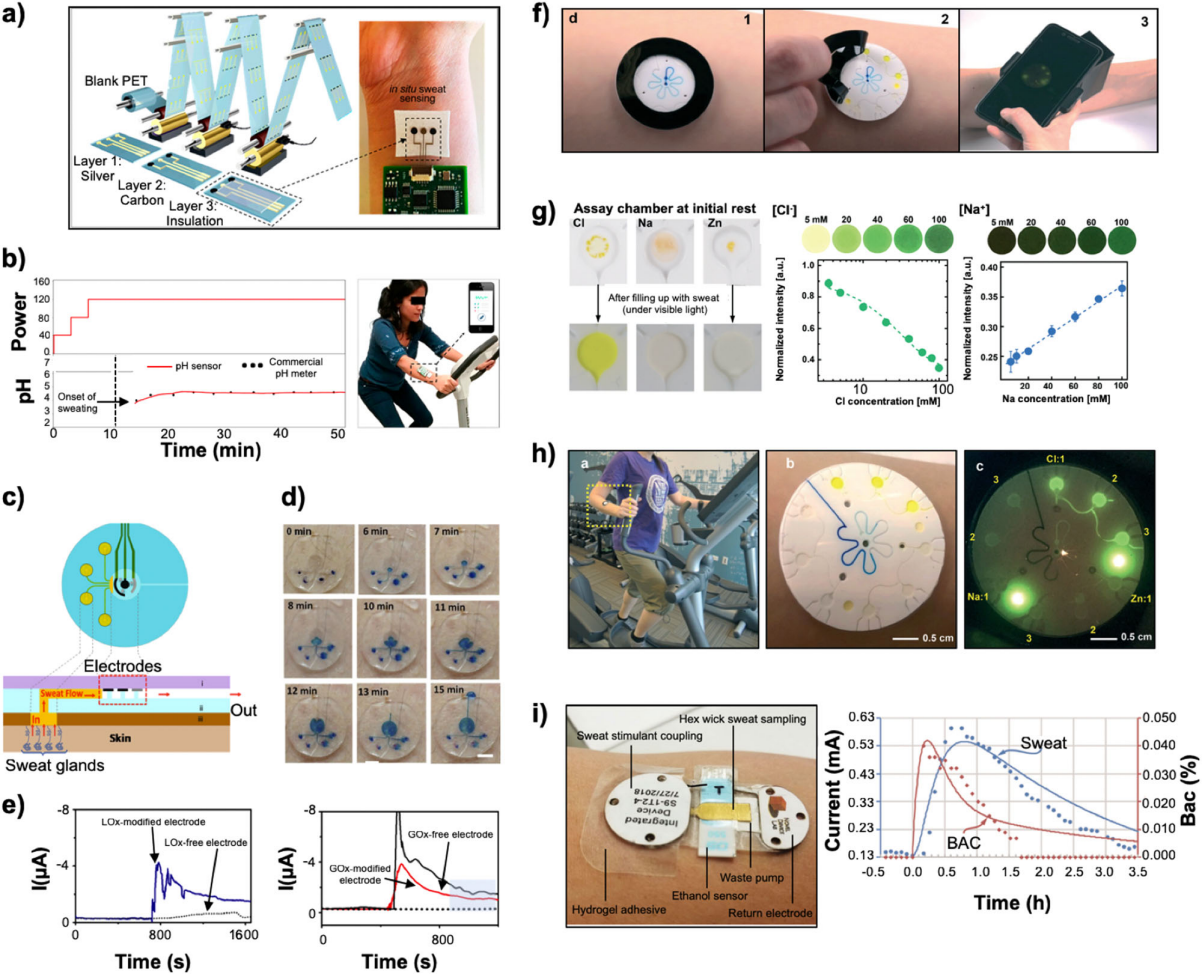
Wearable sensors to monitor the biochemical status of the athlete by detecting biomarkers from eccrine sweat. **a** R2R gravure manufacturing of electrochemical sensors on PET substrates. **b** Real time, in situ measurement of sweat pH from the sensor depicted in panel (**a**). **c** Schematic of the microfluidic sweat collection device. Top-down and cross-sectional views are provided. **d** Photographs depicting the time needed to fill the microfluidic reservoir from panel (**c**) using an optimized four-inlet design when sweat is generated during nonstationary conditions. **e** Continuous lactate and glucose monitoring via the Lox and GOx-modified electrodes from panel (**c**) on a healthy subject. **f** Protocol for performing a fluorometric assay using a microfluidic device to detect zinc, sodium, and chloride levels: (1) collecting sweat using a skin-interfaced microfluidic device, (2) peeling away the black shield, and (3) capturing a photo of the device using a smartphone interfaced with the device with an optics module. **g** Fluorescence images of the detected analytes from the microfluidic device detailed in panel (**f**) and the dependence on fluorescence intensity on concentration. Images of the microreservoirs for the assays before (upper) and after (lower) filling with sweat collected under visible light illumination. Changes of the fluorescence and its normalized intensity are shown at various concentrations and depicted for sodium and chloride. **h** Subject wearing the microfluidic device from panel (**f**) during testing. Photographs of the device without the black shield after sweat collection is shown under visible light and under blue light emitted by a smartphone. **i** After the patch is applied, sweat stimulation involved the iontophoretic delivery of carbachol. Sweat is picked up from the skin by the hex-wick and transported to the sensors to measure ethanol concentration and then transported onto the waste pump. In vivo test data carried over 3.5 h on a subject is shown. The ethanol bolus occurred at the start time and only thirty minutes of sensor results are depicted previous to the ethanol bolus. Figures were reproduced with permission from Bariya et al.^66^ (**a**, **b**), Martín et al.^67^ (**c–e**), Sekine et al.^68^ (**f–h**), and Hauke et al.^23^ (**i**)

Researchers fabricated epidermal polymeric electrodes for the individual or combinatorial detection^44,55^ of lactate,^56^ sodium,^57^ potassium,^58^ glucose,^59,60^ cortisol.^61^ As previously mentioned, Gao et al.^44^ fabricated an epidermal sensor on a PET substrate for the concurrent and continuous detection of sodium, lactate, potassium, glucose, and skin temperature. The sensor for skin temperature compensated for the temperature dependence of the enzymatic reactions. The flexible electrode was connected to a corresponding module for signal processing and subsequent wireless transmission to a Bluetooth device. The flexible nature of the device allowed it to be worn around the wrist (analogous to that of current commercialized wrist-based wearables) to provide direct contact with the sweat on the skin surface. The work filled the gaps between signal processing, filtering, and amplification for the real-time wireless transmission of analyte concentrations during stationary and ambulatory conditions. While such devices can flex (low bending stiffness, excellent utility at low-bending radii), they cannot stretch (inelastic, do not have a low modulus, and do not have the capacity to account for large strain deformations).^62^ As such, disparity between the nonstretchable mechanics of the electrode coupled with the stretchable mechanics of the skin can lead to electrode delamination especially during high-acuity sporting activities.^62^ Work to develop electrochemical biosensors into elastic forms is now focused on stretchable functional materials such as carbon nanomaterials^63^ that can be screen printed onto elastomeric substrates. Abellan-Llobregat^64^ reported on the fabrication of a printable and highly stretchable device based on platinum (Pt)-decorated graphite for sweat glucose detection. The electrode measured the reduction of hydrogen peroxide by chronoamperometry using glucose oxidase immobilized on Pt-decorated graphite. This device was applied on human perspiration samples and demonstrated a strong correlation between glucose concentration in perspiration and glucose concentration in blood, as measured via a commercial glucose meter. In a proof-of-concept study, a printed flexible tattoo-based glucose sensor was fabricated for glycemic monitoring.^65^ The device utilized reverse iontophoretic extraction of interstitial glucose and an enzyme-based amperometric biosensor. In vitro studies using the biosensor demonstrated a linear response toward physiologically relevant glucose levels with negligible responses from electroactive species. The device was applied on human subjects during eating to measure glycemic levels. Results showed that the sensor correlated with that of a commercial glucose meter. The preliminary study suggested that the tattoo-based iontophoresis-sensor platform could be efficacious for diabetes management and could be relevant to monitor biomarkers from biofluids indicative of human performance. In another study, Koh et al.^55^ developed a closed microfluidic system that directly harvested sweat from the pores to measure lactate, glucose, hydronium ions (pH), and chloride. The microfluidic system was comprised of a bottom polydimethylsiloxane (PDMS) layer of 500 µm, imprinted with the necessary geometry (uniform depth, 300 µm), and filled with reagents for colorimetric analysis. The flexible and stretchable sensor adhered to multiple locations on the body without chemical or physical irritation by using biocompatible adhesives, soft device mechanics, and water-tight interfaces. The device routed sweat to the four channels to permit the simultaneous detection of the various biomarkers. Furthermore, the device provided the option for wireless interfaces to external devices for image capture and analysis. The device proved to be efficacious when tested on humans during cycling. Such devices could be commercialized and translated for other sporting events such as football or soccer. Further work regarding the miniaturization of such devices or the combination of sensor interfaces for simultaneous measurements along with the ability to mass produce sensors would enhance the utility of this technology for sports. Bariya et al.^66^ developed roll-to-roll (R2R) gravure printed electrochemical electrodes on 150 m flexible PET substrate rolls for the detection of pH, K^+^, Na^+^, Cu^2+^, glucose, and caffeine (Fig. 2a, b). The team utilized inks and electrode morphologies designed for electrochemical and mechanical stability to achieve devices with uniform redox kinetics. The work represented a significant step towards large-scale, low cost fabrication of disposable wearable sensors for applications in sports medicine and health-related applications. In another study, Martín et al.^67^ developed a microfluidic epidermal device for the detection of glucose and lactate (Fig. 2c–e). The device is composed of two soft, conforming PDMS layers, along with a double-sided adhesive layer (Fig. 2c). The first PDMS layer integrates with the electrode system, while the second PDMS layer contained the microfluidic channels (inlets and outlet) and the detection reservoir. The device adhered to the skin sweat pores to route sweat toward the electrochemical sensor while concurrently enduring repetitive mechanical deformation by the wearer. A representative time-lapse analysis of the sweat flow profile within the microfluidic device when applied to the lower back of a healthy volunteer during exercise activity (in the absence of sensing electrodes) was shown over a 15-min period (Fig. 2d). On-body real-time monitoring of sweat lactate and glucose levels was performed on two healthy human subjects during indoor cycling over a 20-min period (Fig. 2e). The continuous monitoring of the amperometric sweat lactate response from the subjects with the LOX-modified flow detector demonstrated an increase of the current signal as the sweat sample entered and filled the detector reservoir. The same trend was seen when measuring glucose levels using GOX-modified electrodes. The trend in measuring glucose levels from eccrine sweat matched that of blood glucose values; however, the need to prevent sample contamination remains the next step for long-term non-invasive glucose measurements utilizing such technology.

#### Translating sweat sensor technology for sports

Recently reported wearable devices for eccrine sweat analysis offer promising approaches utilizing microfluidics (Fig. 2f–h)^68^ or iontophoresis (Fig. 2i)^23^ to alleviate hurdles associated with sample contamination and/or sample volume. Microfluidic platforms enable collection of sweat from the skin surface by connecting to eccrine sweat glands from various locations on the body with opportunities to scale the size and form-factor of the device to the application of interest. The ability to extract sweat in this manner for laboratory analysis is crucial but relies on postcollection analysis. Colorimetric assays provide a promising alternative that circumvent the need for electrochemical measurements, associated power supplies, and external hardware for data communication. Sekine et al.^68^ developed a fluorometric approach to detect Na^+^, Cl^−^, and zinc (Zn^2+^) from eccrine sweat captured in a wearable microfluidic device utilizing a smartphone-based fluorescence-imaging module (Fig. 2f–h). Reaction of the probes in microreservoirs with the specified ions lead to changes in the fluorescence excitation intensity, as detected by a smartphone outfitted with an optics module. The ion concentrations determined using this platform on human subjects exercising on an elliptical trainer (sweat Cl^−^, 28–31 mM, Zn^2+^ ~2.5 µM, Na^+^ 35–50 mM) matched those obtained using traditional laboratory methods such as ion chromatography for Cl^−^ (28 mM), ICP-MS for Zn^2+^ (3.6 µM), and atomic absorption for Na^+^ (36 mM). Such technology utilizing microfluidics to measure sweat rate and hydration levels from sweat (indicative of changes in Na^+^ concentration) could provide significant advantages compared to currently utilized sweat patches previously reviewed earlier (Table 5). Building upon previously published work by the Rogers Group, Reeder et al.^69^ developed a waterproof, epidermal, microfluidic wearable device capable of adhering to the skin to capture, store, and analyze sweat while fully underwater. The technologies introduced utilize polymeric materials such as poly(styrene–isoprene–styrene), SIS, for skin-compatible microfluidic platforms to enable low rates of water penetration, water vapor, and water-borne chemistries from the surrounding environments for long-term use (hours). Furthermore, the design of the microfluidic channels prevent contamination from aquatic environments without impeding the flow of sweat to the sensor. The sweat rates in swimmers is lower than that of athletes on land and has been shown to range from 0.33 to 1.6 l/h, depending on the workout intensity and water temperature.^70–72^ Monitoring sweat rates, hydration, and ion concentrations is imperative to tracking the performance and health of athletes in events such as ultra-endurance triathlons. The robust and water-tight bonding to the skin, under extreme conditions, enabled the device to adhere to the swimmer’s body for greater than two hours. Clinical studies demonstrated the ability of the device to measure local sweat chloride concentrations, local sweat loss, sweat rate, and skin temperature during intense physical activity in controlled, indoor conditions and in open-ocean swimming during the IRONMAN triathlon. To the best of our knowledge, the published work by Reeder et al. represents the first example in current literature in assessing the utility of such technologies during high-acuity and high-stress sporting situations.

The weather and outside air temperature play a major hurdle in sweat-sensing technology for sports. For example, an elite-level athlete training in a humid environment would generate large volumes of sweat compared to the same athlete training in a cold climate. Given the small sample volumes generated and captured by wearable sweat sensing devices, how can such technology be useful for athletes in cold-weather environments? We hypothesize that the ability to stimulate sweat via iontophoresis could solve this issue. Hauke et al.^23^ developed and validated a continuous and blood-correlated sweat enzymatic sensor with integrated sweat stimulation to detect ethanol (Fig. 2i). Ethanol was selected because it is 1:1 between sweat and blood due to its lipophilic nature. Sweat stimulation by iontophoresis involved three novel steps discussed in this work. The first part involved membrane isolation of the sweat stimulant from the skin to prevent sweat from diluting out the desired analyte. The second part involved the use of carbachol as the stimulant to enable a steady generation of sweat over a long period of time (hours to days). The third was the use of a sudomotor axon reflex sweating to minimize mixing of the old and new sweat thereby minimizing sample contamination. The continuous nature of the data when tested on two human subjects allowed for analysis of blood-to-sweat lag times that ranged between 2.3 and 11.41 min for the onset of the ethanol signal. Further work is needed to study the operation of the device for 24 h or longer and with a broader range of analytes specifically of interest to sports performance. Nonetheless, the work confirmed that sweat can be stimulated and the desired ions or analytes can be measured in a continuous manner to correlate with that in blood.

Human performance is a function of the physical demands of the sport coupled with the mental acuity exerted on the athlete. In this section we have focused on the former. In the next section, we will focus on the latter, an emerging area where much research and development remain to be accomplished.

### Mental acuity of the athlete to optimize on-field performance

**Fig. 3:**
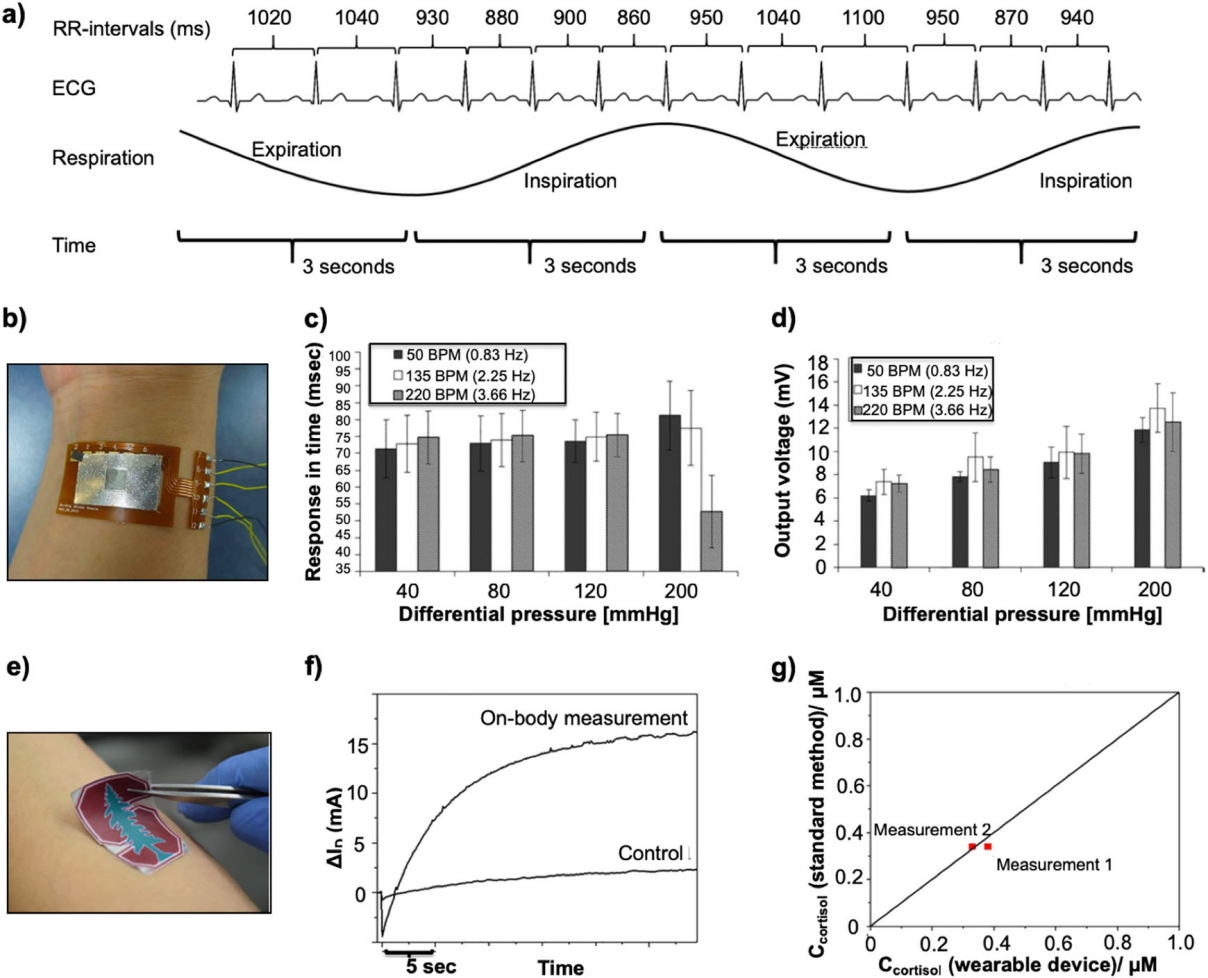
Monitoring the mental acuity of the athlete via measurement of heart rate variability, skin conductivity (galvanic skin response), or biomarkers from eccrine sweat. **a** Schematic illustrating the derivation of heart rate variability from an ECG. The ECG presented herein is depicting respiratory sinus arrhythmia. Heart rate increases thus decreasing the time between successive RR intervals during inhalation and exhalation. The change in time between successive RR intervals is called heart rate variability, expressed in ms. Short heart rate variability is indicative of high-stress levels whereas long heart rate variability is indicative of a calm period. **b** Human stress monitoring patch affixed to a human wrist (**c**) Performance of the pulsewave sensor from panel (b) for varying differential pressure of heart beat depending on the heart rate of 50 BPM, 145 BPM, and 220 BPM as a function of the change in time. **d** Performance of the pulsewave sensor from panel (**b**) for varying differential pressure of heart beat depending on the heart rate of 50 BPM, 145 BPM, and 220 BPM as a function of output voltage. **e** Image of an epidermal sensor applied to the forearm of a healthy volunteer to detect cortisol levels from eccrine sweat. **f** Real-time response of the molecularly selective and control devices after completion of physical exercise. The cortisol response was recorded using the output measurement and the data were represented as a change of drain current vs. time at a low voltage. **g** The data demonstrated a good correlation with standard cortisol ELISA methods for cortisol detection with an RSD of 5% for the two measurements. Figures were reproduced with permission from Firstbeat Technologies^80^ (**a**), Yoon et al.^74^ (**b–d**), and Parlak et al.^111^ (**e-g**)

Monitoring stress levels can help manage the well-being of an athlete through a season.^73^ A stress reaction triggers the release of hormones such as epinephrine and cortisol. There are three primary methods to monitor stress levels for athletes: (1) self-reporting (current method), (2) multimodal physiological analysis, and (3) body-fluid analysis^74^ (Table 6). The self-reported method is disadvantageous to measure human stress levels due to the lack of standards for stress status and the inability to assess the wellness and mental acuity of the athlete in a real-time manner.^75^ We focus our attention on monitoring stress levels via the latter two methods and focus our attention on the application of wearable sensors to measure HRV, skin conductivity, and biomarkers such as Cortisol and Orexin A from eccrine sweat (Fig. 3).

**Table 6:** Comparative analysis of various stress measures to evaluate the mental acuity of the athlete

Measure	Advantages	Limitations	Utility of CM for HPA
Stress–response questionnaire	• Easy to perform, • Large sample sets possible • Cost efficient	• Subjective measures • Lack direct link to stress response • Time intensive process	No. Teams do not have the time to conduct such questionnaires constantly
Physiological interviews	• More personable than a generic questionnaire • Higher likelihood of detailed analyses	• Time consuming process • Need for trained interviewees	No. Teams do not have the time to conduct such questionnaires constantly
Heart rate variability	• Objective and non-invasive method to assess the ANS	• Not easily interpretable as stress varies with time • No standard to quantify stress level based on HRV	Yes. Wearable devices exist. Formal clinical studies needed to assess their use-case for sports
Blood pressure	• Noninvasive and objective measurement possible	• Continuous measurements are challenging • Direct link to stress levels have not been formally identified	Yes. Wearable devices exist. Formal clinical studies needed to assess their use-case for sports
Brain Activity (e.g., EEG, neuropriming)	• Noninvasive and objective measure of chronic stress	• Difficulty in measuring long-term. • Very limited use-case in sports.	Yes. Wearable devices exist. Formal clinical studies needed to assess their use-case for sports
Skin conductance	• Noninvasive • Fabrication of epidermal electronics makes this route possible long-term	• Results obscured by eccrine sweat during workout • Limited utility during physical activity	No. Currently there are no commercial sensors (sampling of devices exists in literature)
Biomarkers (e.g., Cortisol, Orexin A)	• Ability to detect key biomarkers indicative of stress from bodily fluids	• Current technology is relatively immature • Scientific results are mixed • Sample analysis often requires laboratory equipment	No. Currently there are no commercial sensors (devices exist based on those in literature)

*CM* continuous monitoring, *HPA* human performance assessment

#### Stress and athlete recovery

Optimal training, well-balanced diet, and recovery form the foundation of success of an athlete. Balancing training intensity as a function of workout schedule or duration with recovery enables athletes to maximize their performance and further the player development process. Overtraining without adequate rest may lead to the overtraining condition, which is characterized by decreased performance and subsequent detriments to long-term health. A systematic objective assessment of an athlete's recovery is crucial to prevent overtraining. Recovery should be constantly evaluated for optimizing the stress exerted on the body and for avoiding both over- and undertraining when seeking the most efficient training regimen.

The human nervous system is made up of the central and peripheral nervous system with the latter comprised of two divisions, the voluntary and autonomic system (ANS). The voluntary nervous system is concerned mainly with movement and sensation whereas the ANS controls functions over which an individual has less conscious control (e.g., cardiovascular system). Stress reactions are the way the human body tries to cope with the demands of the surrounding environment. Positive stress can be considered as “getting the job done”. Negative stress causes emotions and reactions deleterious to the human body. A stress reaction causes the activation of the ANS and the production of stress hormones along with an increased HR and an increased force of heart contractions.^76^ While there currently lacks a formal definition of stress in scientific literature, we postulate that stress can be physiologically characterized by a reduced recovery of the neuroendocrine reaction and sympathetic dominance of the ANS function.^77^ Recovery can be characterized as parasympathetic dominance. The magnitude of the neuroendocrine response reflects the metabolic and physiological demands required for a given activity.^77^ In other words, the body should adapt to the demands put upon it in various situations. Problems arise when the body is not able to adapt to changing demands. The body needs to utilize more sympathetic activity during stressful periods, active working, or during physical activity. Parasympathetic activity should be dominating the ANS activity during the sleep period. Monitoring such activity in a noninvasive manner provides tremendous insight into the physiological and mental status of an athlete to maximize performance, health, and safety.

#### Heart rate variability

The ANS plays a major role in modulating the HR. The heart contracts according to an automatic, or intrinsic, rhythm regulated by the sinus node. The normal resting HR in a sitting position ranges between 60–80 beats/min due to the sympathetic and parasympathetic nervous systems, hormonal factors, and reflexive factors.^78^ Fluctuations in HR caused by respiration are referred to as respiratory sinus arrhythmia (RSA).^79^ Specifically, HR increases during inspiration and decreases during expiration. The fluctuation in the time between successive heartbeats is called HRV (Fig. 3a).

The sympathetic and parasympathetic nervous systems maintain cardiovascular homeostasis by responding to beat-to-beat perturbations that are sensed by baroreceptors and chemoreceptors.^80^ During physical activity, the parasympathetic activity is first withdrawn and then sympathetic nerve activity is augmented to meet the metabolic demands of the behavior.^81^ Concurrently, HR increases and HRV decreases with increasing exercise intensity. Monitoring HRV provides useful knowledge for observing the interplay between the sympathetic and parasympathetic nervous systems, and is reflective of ANS activity.^82^ Thus, a low HRV (short-time interval) is suggestive of a stressful period whereas a prolonged HRV (long-time interval) is suggestive of a calm period. HRV remains the most common method to determine the stress. The training status of athletes may affect HRV.^83,84^ Overtraining is caused by long-term stress or exhaustion due to imbalance between training, other external/internal stressors, and recovery. HRV is also affected by the training load of individual exercise sessions; the higher the training load, the lower the HRV after exercise.^85^ Thus, HRV is context dependent so the overall environment the athlete is in must be factored in prior to monitoring and subsequent diagnosis.

The quantification of ANS function is possible by calculating parameters pertinent to HRV according to time-domain, frequency-domain, and nonlinear analysis of consecutive RR intervals of an ECG waveform.^86^ These parameters represent various components of the sympathetic and/or parasympathetic system of the ANS. For example, the high-frequency component derived by the frequency domain analysis denotes the parasympathetic activity.^87^ Successful derivation of these HRV parameters is dependent on the recording quality, the subject’s activity during the recording, the removal of artifacts, the detection of arrhythmic beats, and the recording duration (seconds to days). ECG monitoring (and subsequent derivation of stress from HRV levels) via the use of wearable sensors poses several challenges for athletics.^88^ Firstly, surface EMG, increased electrode impedance, respiration induced baseline drift, and electrode contact movement can cause noise and motion artifacts.^88^ Secondly, heterogeneity in the QRS complex often poses challenges to identify the RR interval.^89^ Lastly, a reported drawback in most ECG-based wearable devices that do not record standard ECG derivations is their inability to distinguish some arrhythmias and ectopic beats.^90^ The recent FDA clearance of the ECG sensor on the Apple Watch 4 could enable a shift in this regard; however, clinical validation of such technologies is greatly needed to negate concerns posed by clinicians regarding its efficacy.^91–93^ A recent systemic review sought to investigate if wearable devices provide an accurate and reliable measure of HRV parameters during rest and exercise.^94^ Eighteen studies were selected: sixteen of them utilized ECG–HRV technology and two of them utilized photoplethysmography-pulse rate variability (RV) technology. All of the studies looked at the accuracy of wearable devices in RV detection during rest, while only eight of them evaluated their accuracy during exercise. The correlation between ECG-derived HRV and the wearable RV was validated during rest but declined as exercise intensity was increased. The study concluded that wearable devices such as the BlueLeza HRM Blue, Carre Technologies Hexoskin, Garmin wrist-watches, Polar H7 HR Monitor, VivaLnk Vital Scout Patch, and Whoop Strap 2.0 may provide a promising alternative solution for measuring RV; however, more robust studies in nonstationary conditions are needed specifically with larger subject populations to fully derive their clinical utility for sports.

#### Skin conductivity

Multimodal physiological monitoring permits the continuous and consistent detection of stress.^74^ The human ANS is responsible for the changes in stress levels when excited by various stressors. Skin temperature, skin conductance, and arterial pulsewave signals among other ANS responses are necessary for a multimodal physiological data analysis.^95^ These signals are categorized into acute or chronic stress. A negative correlation exists between peripheral skin temperature and chronic stress levels,^96,97^ while a positive correlation exists between skin conductance on the palm and volar wrist with chronic and acute stress levels.^95^ To characterize stress, arterial pulsewave signals are transformed into HRV, which represents the chronic stress level and an individual’s stress vulnerability.^98^ Research-oriented stress monitoring devices are bulky and must be worn or carried for everyday use. Thus, development of epidermal or wrist-based sensors for multimodal physiological data detection and analysis would greatly aid in advancing this field. Researchers have developed and fabricated an epidermal sensor for the multimodal physiological data analysis of stress via measurement of skin temperature, skin conductance, and arterial pulsewave signals (Fig. 3b–d).^74^ The stress patch measured skin temperature with a sensitivity of 0.31 Ω/°C, skin conductance sensitivity of 0.28 μV/0.02 μS, and a pulse wave response time of 70 ms.^74^ The patch categorized the four types of human emotions (surprise, anger, stress, and sadness) based on a singular vector machine algorithm. While beyond the scope of the study, we hypothesize that such technology could have utility to measure the wellness of athletes in a real-time manner. Clinical testing in this regard is necessary and currently lacking.

The galvanic skin response, GSR, (otherwise referred to as the electrodermal activity, EDA) refers to changes in sweat gland activity that are reflective of the intensity of one’s emotional state. Emotional levels change in response to the environment—if an event is deemed scary, threatening, joyful, or emotionally relevant, then the subsequent change in GSR is reflective in the emotional response. This change increases eccrine sweat gland activity. Thus, measuring biomarkers from eccrine sweat using wearable sensors could be useful in measuring stress levels in a noninvasive manner to assess the mental acuity of athletes real-time. Furthermore, the development of sensors capable of measuring both GSR/EDA and biomarkers from eccrine sweat indicative of stress and fatigue could be extremely useful in providing a holistic measure of the mental and physiological status of the athlete.

#### Biomarkers indicative of stress levels

Prior work to measure stress-related biomarkers has utilized high-performance liquid chromatography (HPLC),^99^ enzyme-linked immunosorbent assay,^100^ radioimmunoassay kit,^101^ or a HPLC mass spectrometry (MS) system.^102^ In addition, EEG has been utilized as a noninvasive means to measure stress levels by placing electrodes on the human scalp which measure oscillations of the brain’s electric potential.^103,104^ Collaborative research undertakings, such as the Online Predictive Tools for Intervention in Mental Illness (OPTIMI), have recorded EEG and ECG activity, voice analysis, electronic diaries, and cortisol sampling to monitor an individual’s mental state.^105^ These existing methods are limited for use in sports applications due to the significant cost and technical expertize required. Thus, measuring stress-indicative biomarkers in a non-invasive manner would greatly advance this field.

Cortisol is a steroid hormone secreted from the adrenal glands in response to stress as a product in the hypothalamic–pituitary adrenal pathway.^102^ Cortisol is responsible for maintaining homeostasis in the body via the regulation of neural, immune, cardiovascular, metabolic, and endocrine systems.^102^ Cortisol has been measured in human blood,^106^ serum,^107^ urine,^107^ saliva,^107,108^ hair,^109^ interstitial fluid,^110^ and most recently eccrine sweat.^111^ In a recent study, Jia et al. sought to further understand and quantify cortisol in human eccrine sweat by utilizing LC–MS.^102^ The study detected one isomer that had a similar hydrophobicity, retention time, and fragmentation patterns to that of cortisol found in eccrine sweat. Prior studies have shown that the levels of certain molecular markers in eccrine sweat are comparable to those found in human plasma. Marques-Deak et al.^112^ compared baseline levels of cytokines, such as IL-1α, IL-1β, IL-6, TNF-α, IL-8, and TGF-β in plasma and eccrine sweat, and showed that the measured cytokine levels were comparable to that of circulating levels in plasma.^112^ Prior work using biosensors to measure stress and cognition has focused on the measurement of a wide array of biomarkers (10^−6^–10^−12^ M) such as Orexin-A,^113^ cortisol,^110^ dopamine,^114^ neuropeptide Y,^115^ and interleukin-6 (IL-6).^116^ Parlak et al. developed a multifunctional layered wearable organic electrochemical sensor for the non-invasive detection of cortisol from eccrine sweat (Fig. 3e–g).^111^ The team integrated an electrochemical transistor and a biomimetic polymeric membrane, which permitted the detection of cortisol. The sensor, combined with a microcapillary channel array, was integrated in the sensor thereby providing precise sample delivery to the sensor interface. Ex situ testing, performed by spraying artificial sweat with increased cortisol concentrations on the forearm, and real-time testing during exercise, demonstrated the utility of the device to measure cortisol (Fig. 3e–g). The study suggested that the ability of the sensor to be adapted for the detection of other molecules (noncharged biomolecules and hormones) from eccrine sweat.

Orexin peptides and their corresponding receptors in the brain contribute to autonomic control, attention, feeding, memory, sleep, and stress.^113^ Measuring serum levels of Orexin A can aid in predicting mood and cognitive performance in athletes.^113^ Noninvasive measurement of several neuropeptides utilizing functionalized antibodies or peptide-decorated semiconductors has been reported.^117,118^ Hagen et al. utilized interdigitated zinc oxide field effect transistors (ZnO FETs) to detect Orexin A by binding a bifunctional peptide to both the ZnO semiconductor and the neuropeptide.^113^ The binding was transmitted to an electrical signal and the sensor selectivity was able to detect concentrations of approximately 100 aM in water, 10 fM in filtered human saliva, and 1 nM in filtered fetal bovine serum. The sensor platform demonstrated the potential of an FET device to measure a wide array of biomarkers in complex biofluids; however, development of improved sensitivity and stability of biosensors is required for the real-time detection of such biomarkers.^113^

The utilization of wearable sensors to measure the aforementioned biomarkers represents a significant opportunity to monitor stress levels in athletes.^119^ However, a majority of the devices presented in both scientific and commercial literature are not yet validated in clinical studies for real-time assessment of human performance. Companies such as VivaLnk, Sentio Feel, and Interaxon Muse have devices that can monitor and detect stress levels in real-time via measurement of electrodermal activity, HRV, or utilizing signal processing methods from EEGs. In addition, devices such as those by Halo Neuroscience can potentially improve cognition during athletic training sessions by utilizing neuropriming to increase the excitability of motor neurons.^120^ Research showed that transcranial direct current stimulation at low currents (e.g., <2 mA) applied over the scalp induced changes in brain excitability for an extended duration to result in synaptic and nonsynaptic functional changes.^121,122^ Clinical trials by Halo Neuroscience have shown that this effect can significantly improve player performance and training.^120^ Leveraging the data acquired from such devices, analytical platforms can be implemented to correlate biomarker concentrations with stress levels as a function of time to help an athlete eliminate costly errors during high-acuity situations. In addition, training regimens can be developed to expedite player development process and enable the athlete to reach their peak performance at a faster rate. The need to develop multi-modal sensors integrating various parameters such as biochemical markers, HR, HRV, sleep, and/or skin conductivity (as a function of GSR) to accurately measure stress levels coupled with their clinical validation in nonstationary conditions is greatly needed to enable athletes to maximize their performance, recovery, and health. Lastly, there remains an unmet medical need to differentiate between physical and mental (psychological) stress to understand the physical and mental demands of the athlete. We hypothesize that the development of multimodal sensor technology could enable such distinctions to be made via an array of factors that are indicative of various stressor types.

## Opportunities and future outlook

The emergence of IC fabrication strategies, flexible electronics, device design, and e-garments has revolutionized the development of soft electronics and biological and chemical sensor technologies toward advancing sports medicine.^123–127^ The wearables field has recently seen a variety of devices for detecting position and motion, coupled with the emergence of clinical studies to assess their validity in professional or collegiate sports teams. However, as previously mentioned, devices seeking to measure biosignals, biomarkers, and biomechanical parameters are challenged by the device technology itself and systems level issues associated with data analytics/data mining.

On the device side, first and foremost, there remains a need to develop the sensing technology, and packaging approaches designed for robust and easy-to-wear systems that increase detection sensitivity as well as improve the signal to noise ratio, specifically at the interface between soft sensing components and rigid electronics. Second, reducing the overall power of the device is crucial to moving these devices past the initial prototype stages. Third, moving toward Application Specific Integrated Circuit (ASIC) technologies to consolidate electronics and reduce power consumption will help play a role in their miniaturization and inclusion in garments or other modalities for athletics. Lastly, to lower cost, it will be crucial to scale sensor production into volume via panels or roll-to-roll manufacturing platforms, as previously reviewed in the work from Bariya et al.^66,128^

**Fig. 4:**
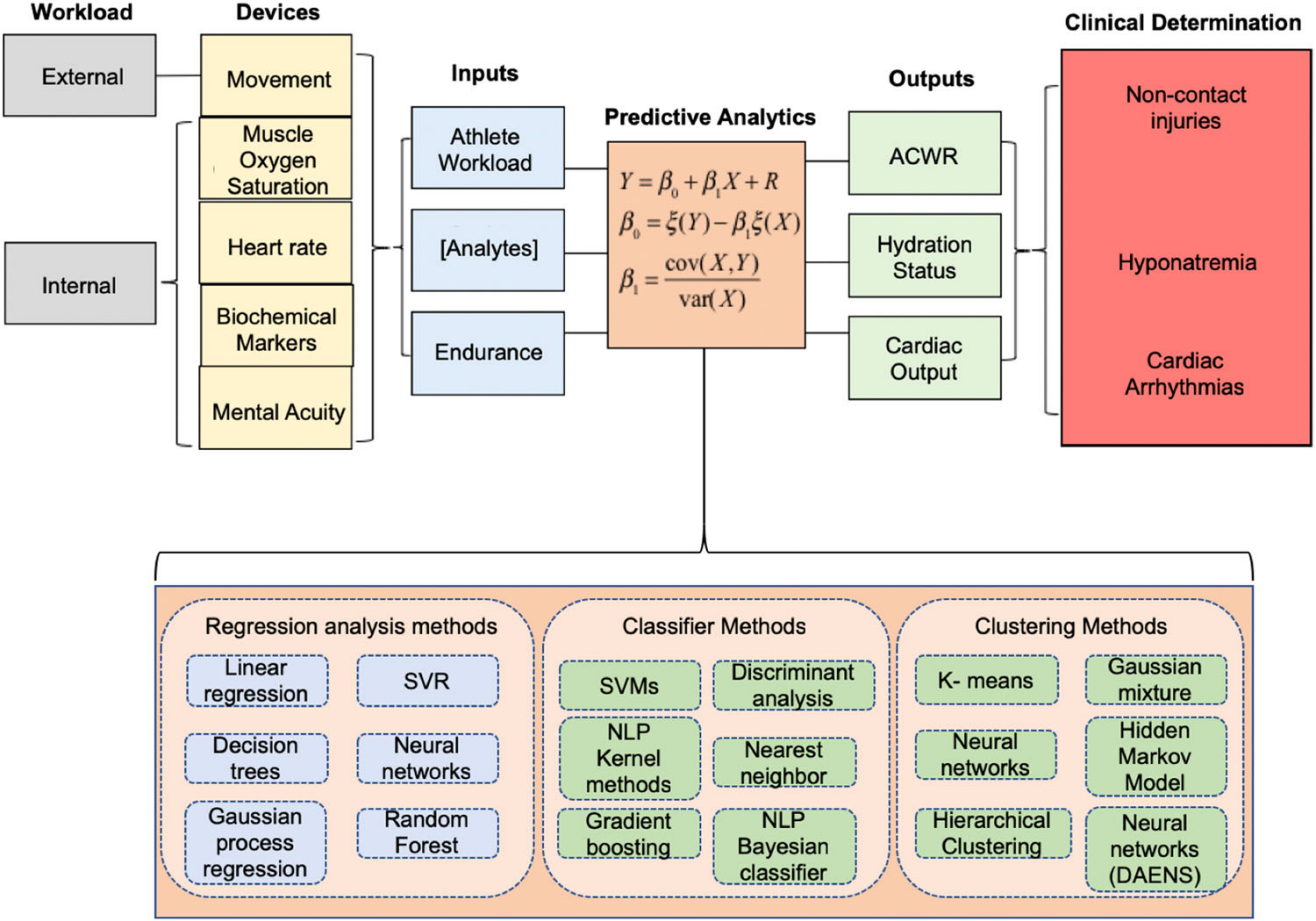
Emergence of machine learning could heighten the translational utility of wearable sensor technology for sports. Data acquired from wearable sensors can be inputted into machine learning models to predict athlete performance, likelihood of suffering a noncontact injury, inform hydration status to alleviate soft-tissue injuries, or accurately diagnose cardiac arrhythmias

On the systems side, there remains a need to improve analytics and data mining techniques to translate the acquired data from these sensors into actionable protocols for the athletes. The primary function and role of data mining for biomedical devices and sensors, includes: (1) data acquisition via the wearable sensor, (2) data transmission from the athlete to team trainer, (3) data integration, (4) data storage, and (5) data security and privacy.^129^ Consideration of such issues has led to improvements in data filtering, signal processing, and noise removal. To solve such problems, data mining techniques such as wavelet analysis for artifact reduction and data compression, rule-based methods for data summary and transmission, and Gaussian processing for secure authentication have been implemented.^129^ There remains an unmet medical need to further develop acute-to-chronic workload ratio models from wearable devices to hone in on ranges indicative for the sport of interest. Current models focus in on only select sports such as Australian Football, Cricket, or Rugby. The emergence of machine learning^130,131^ and artificial intelligence^132^ toward this translation is critical for the growth of the wearables field (Fig. 4). Kitman Labs utilizes machine learning platforms and motion technology to diagnose and uncover an athlete’s unique stress response and various movements.^133^ Clinical studies utilizing wearable sensors for sports medicine can enable researchers to gain access to a wide array of data sets thereby allowing the training of analytical models to accurately, efficiently, and precisely predict athlete injuries based on workload profiles to ultimately translate the acquired sensor data into actionable protocols for sports-medical personnel. With such advancements, one could envision a platform which translates these physiological parameters and biomarkers to provide key medical personnel a real-time status of how the athlete is performing and advise the trainer and team physician as to the necessary recovery protocol for the individual. Open-source platforms such as the American Heart Association Precision Medicine Platform (powered by Amazon Web Services, AWS)^134^ to allow researchers to upload data from clinical studies will be key to advancing the translational utility of this field for sports medicine. Ultimately the goal of wearable sensors for sports medicine is to develop a multimodal, nonintrusive device towards the non-invasive, continuous, and combinatorial measurement of both physiological parameters and biomarkers. Succeeding at each of these current technological roadblocks both from the device and systems side will enable the translation of this technology to greatly aid team physicians and sports-medicine trainers to efficiently and accurately monitor and tailor treatment plans to maximize player performance and minimize injury.
